# Physical Inactivity and Possible Sarcopenia in Rural Community Daycare Stations of Taiwan: A Cross-Sectional Study

**DOI:** 10.3390/ijerph19042182

**Published:** 2022-02-15

**Authors:** Yu-Zu Wu, Ching-Hui Loh, Jyh-Gang Hsieh, Shinn-Zong Lin

**Affiliations:** 1 Department of Physical Therapy, College of Medicine, Tzu Chi University, Hualien 97004, Taiwan; 2 Center for Aging and Health, Hualien Tzu Chi Hospital, Buddhist Tzu Chi Medical Foundation, Hualien 97002, Taiwan; twdoc1960@gmail.com; 3 Aging and Health Research Center, National Yang Ming Chiao Tung University, Taipei 11221, Taiwan; 4 Department of Geriatric Medicine, National Yang Ming Chiao Tung University School of Medicine, Taipei 11221, Taiwan; 5 School of Medicine, Tzu Chi University, Hualien 97004, Taiwan; jyhgang@gmail.com; 6 Department of Family Medicine, Hualien Tzu Chi Hospital, Buddhist Tzu Chi Medical Foundation, Hualien 97002, Taiwan; 7 Department of Neurosurgery, Hualien Tzu Chi Hospital, Buddhist Tzu Chi Medical Foundation, Hualien 97002, Taiwan; shinnzong@yahoo.com.tw

**Keywords:** daycare station, physical inactivity, physical performance, possible sarcopenia, prevalence, rural community

## Abstract

Physical inactivity and possible sarcopenia pose a challenge for long-term care, especially in rural areas. We aimed to examine the prevalence of and associated factors for physical inactivity and possible sarcopenia in rural community daycare stations. A total of 275 adults aged 55–98 years (75% women) were recruited from all 11 rural community daycare stations in Northern Hualien, Taiwan. Physical inactivity was defined as less than 150 min/week of moderate-intensity aerobic physical activity. Possible sarcopenia was defined according to the Asian-specific criteria from 2019. Multiple linear and logistic regression analyses were used to determine associated factors for physical inactivity and possible sarcopenia. The prevalence of physical inactivity and possible sarcopenia was 29.1% and 68.7%, respectively. About 86.8% of possible sarcopenia were ascribed to poor five-times-sit-to-stand performance. After adjusting for covariates, poor lower-limb muscle function, e.g., slow gait speed, was associated with possible sarcopenia and physical inactivity. However, physical inactivity was not independently associated with possible sarcopenia (adjusted odds ratio 1.95, 95% confidence interval 0.88–4.30, *p* = 0.100). Our results indicated that individuals with poor lower-limb muscle function were more likely to have possible sarcopenia and physical inactivity. Improving lower-limb muscle function would be a priority task in rural community daycare stations.

## 1. Introduction

In Taiwan, the pace of population aging is faster than in Japan, Europe, and the US [1]. In response to the long-term care (LTC) needs of a fast-growing older population, the Taiwanese government has been developing community daycare stations which put aging-in-place values into practice by delivering an integrated community-based primary healthcare and preventive service [2,3]. Community daycare stations have been suggested by the Asian Working Group for Sarcopenia in 2019 (AWGS 2019) to be amenable to implementing an integrated LTC service to prevent or delay disability, especially for people who are physically inactive or at risk of sarcopenia [4]. In Taiwan, this setting is of significance especially in rural areas with limited and imbalanced healthcare resources.

Sarcopenia is characterized by an age-related decline in muscle mass and muscle function [4]. Many studies have indicated that sarcopenia was associated with substantial adverse health outcomes, such as falls, fractures, disability, and loss of independence [5,6,7,8]. The prevalence of sarcopenia varied across the studies, ranging from 5.5% to 46% among the Asian population [4,9,10,11,12,13]. A high prevalence of sarcopenia has been reported to confer an enormous financial and medical burden on both caregivers and the public health infrastructure [14]. Although sarcopenia often develops insidiously, it can be clinically diagnosed by measuring levels of muscle mass. However, measuring muscle mass is not always available in primary healthcare or community settings because a reliable and validated diagnostic tool is not easily accessible [4].

Considering that the role of muscle strength in age-related muscle wasting is more dominant than muscle mass, and that low muscle strength on its own is adequate for starting an integrated care planning, the AWGS 2019 proposed a new concept of possible sarcopenia, defined as low muscle strength and/or low physical performance, as an initial assessment in the early stages of sarcopenia development for primary healthcare or community-based health promotions among the Asian population [4]. Recently, a cross-sectional study has indicated that possible sarcopenia demonstrated excellent diagnostic accuracy for definitive sarcopenia [15].

The epidemiological information regarding possible sarcopenia is scarce. To date, there have been six cross-sectional studies in Japan, Korea, China, and Taiwan that explored the prevalence of possible sarcopenia among the Asian population using the updated AWGS 2019 criteria [9,10,13,16,17,18]. Of these, only three cross-sectional studies explored the associated factors for possible sarcopenia [9,16,17]. Across the studies with respect to different settings and populations, the prevalence of possible sarcopenia varied from 2.9% in Japan [17] to 98.1% in Taiwan [18]. Since possible sarcopenia is still a new concept, more information regarding possible sarcopenia could help provide a more effective delivery of services to those in need of LTC, especially at rural community daycare stations.

Regular physical activity has been shown to have a positive impact on the health status of older adults [19,20], while physical inactivity is considered to be one of the important factors contributing to sarcopenia [21]. According to the World Health Organization (WHO) guidelines [22], the amount of physical activity should be at least 150 min of moderate-intensity aerobic physical activity per week, and those who cannot meet the criteria are considered to have physical inactivity or sedentary behavior. A systematic review indicated that in most studies, 40–80% of older adults are physically inactive [23]. Although increasing evidence suggests that physical inactivity can increase the risk of sarcopenia in older adults [11,24,25,26], the information regarding the relationship between physical inactivity and possible sarcopenia is limited. At present, there have been two Asian cross-sectional studies that reported the relationship between physical inactivity and possible sarcopenia, but the findings are inconclusive and controversial [9,17].

To our knowledge, few studies have explored the relationship between physical inactivity and possible sarcopenia, especially at rural community daycare stations. In this work, we hypothesize that physical inactivity may positively correlate with possible sarcopenia. Thus, we conducted a cross-sectional study aimed at determining the prevalence of and associated factors for physical inactivity and possible sarcopenia, as well as exploring the correlation between physical inactivity and possible sarcopenia in a sample of adults at rural community daycare stations.

## 2. Materials and Methods

### 2.1. Study Design, Setting, and Participants

This was a cross-sectional study conducted at rural community daycare stations in Northern Hualien, a region located on the east coast of Taiwan. In these areas, there were a total of 11 daycare stations that provided integrated LTC services 5 days a week, including shuttle bus transportation, congregational meal services, public health education, leisure activities, and simple physical and cognitive activities, all of which were conducted under the assistance of trained volunteers from the community [2,3]. People who are frail or have mild disabilities are eligible for availability of these LTC services at community care stations [27].

**Figure 1:**
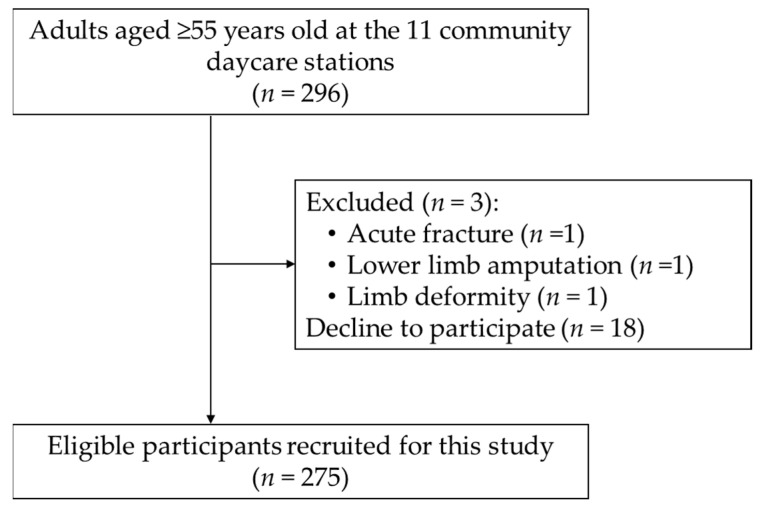
Flow chart of study participants.

All people who were availing of LTC services at the 11 rural community care stations were recruited and screened for inclusion and exclusion criteria via a face-to-face interview. The inclusion criteria were (i) age ≥ 55 years old; (ii) able to follow simple instructions; (iii) availing of LTC services at community care stations at least 4 days per week; (iv) willing to provide written informed consent. The exclusion criteria were acute diseases, fractures, or surgeries within the past 6 months, lower limb amputation, and limb deformity limiting mobility. Figure 1 shows the flow chart of study participants. The study protocol was approved by the Research Ethics Committee of the Hualien Tzu Chi Hospital, and all participants provided written informed consent for study.

### 2.2. Assessment of Possible Sarcopenia

According to the AWGS 2019 criteria, possible sarcopenia was defined as low handgrip strength (<28 kg for men and <18 kg for women), poor five-times-sit-to-stand (5xSTS) performance (≥12 s), or both [4]. Handgrip strength was assessed in the seated position (with the arm 90 degrees to the side) by using a Jamar hydraulic hand dynamometer (Model 5030; Sammons Preston, IL, USA), which was recommended by the AWGS 2019 [4]. Participants were asked to maximally squeeze the handle twice for each hand with a 30 s rest between trials. The maximum value of the twice trials across both hands was taken [4]. The 5xSTS performance was assessed twice by asking participants to stand up and sit down five times as quickly as possible, while their arms were folded across their chest. The shorter time taken from the two trials was recorded for further analysis. The 5xSTS test has been considered a highly valid and reliable tool for the evaluation of lower-limb muscle function [28].

### 2.3. Assessment of Physical Inactivity

Physical activity levels were assessed through a series of questions that required participants to recall physical activity behaviors over the past 6 months. These questions included (a) “Did you participate in physical activity?” If participants answered “yes,” they were further asked (b) “What type of physical activity did you participate in?” and further details were obtained regarding the intensity, frequency, and duration of physical activity. According to the WHO Guidelines on Physical Activity and Sedentary Behaviour [22], participants were considered to have physical inactivity if they self-reported that their physical activity levels were less than 150 min of moderate-intensity aerobic physical activity per week during the past 6 months.

### 2.4. Assessment of Other Covariates

The covariates were age, sex, body mass index (BMI), comorbidities, a history of falls, and lower-limb muscle function, i.e., gait speed and timed up-and-go (TUG) performance. BMI was calculated by diving body weight (kg) by height (m) squared (kg/m^2^). Comorbidities were self-reported based on physician diagnoses, and included five common chronic conditions in Taiwan, i.e., hypertension, cardiac disease, diabetes mellitus, knee osteoarthritis, and stroke [29]. Participants were considered to have a history of falls if they experienced any fall events over the past 6 months. For gait speed, participants were asked to walk at their usual and comfortable pace along a 10 m walkway. The time to complete the test was computed and averaged over the two trials [4]. For the TUG performance, participants were asked to stand up, walk at a comfortable pace to a cone on the floor 3 m away, turn around, and then return to a seated position in the chair, after which the shorter time over the two trials was determined. Gait speed of <1.0 m/s was considered low for physical performance according to the AWGS 2019 criteria, especially for individuals in acute to chronic health care or clinical research settings [4], and a TUG of >11 s was recognized as at risk of falling for community-dwelling older adults [30]. Evidence suggests that both gait speed and TUG tests are highly valid and reliable tools for the evaluation of lower-limb muscle function [28]. All assessments were conducted by a licensed physical therapist.

### 2.5. Statistical Analysis

Descriptive statistics were used to describe participant characteristics. Continuous variables were expressed as mean and 95 % confidence interval (CI), and categorical variables were expressed as frequencies and percentages. The chi-square test and Student’s t-test were used to determine gender differences in categorical and continuous variables, respectively. Univariate linear and logistic regression models were used to assess the changes in participant characteristics for the continuous age variable. Multiple logistic regression analyses were used to determine factors that were associated with physical inactivity and possible sarcopenia, and data were expressed as odds ratios (ORs) and 95% CI. Associations with *p*-values ≤ 0.2 in the univariate regression analyses were selected as covariates for multiple regression analyses. Listwise deletion was used in statistical analysis of missing data. SPSS version 20.0 (IBM, Armonk, NY, USA) was used for statistical analyses. A probability value of *p* < 0.05 was considered statistically significant.

## 3. Results

### 3.1. Demographic Characteristics

Overall, 296 adults aged ≥ 55 years attended the 11 rural community daycare stations in Northern Hualien, Taiwan, at the time of the study. Of these, 3 ineligible adults were excluded, and 18 adults declined to participate in this study. Finally, a total of 275 participants (75% women) aged 55–98 years (mean age, 71.9 years), with a mean BMI of 26.9, completed data collection (Figure 1).

The participant characteristics and their relationship with advanced age are shown in Table 1, indicating that the prevalence of physical inactivity and history of falls was 29.1% and 31.3%, respectively. According to the AWGS 2019 guidelines, the prevalence of possible sarcopenia was 68.7%, with 39.6% low handgrip strength, 59.6% poor 5xSTS performance, and 41.5% slow gait speed. About 86.8% of possible sarcopenia were ascribed to poor 5xSTS performance, and 57.7% of possible sarcopenia were attributed to low handgrip strength. BMI (*β* = −0.19, *p* = 0.003), handgrip strength (*β* = −0.35, *p* < 0.001), and gait speed (*β* = −0.39, *p* < 0.001) significantly decreased with age. The time required to perform TUG (*β* = 0.26, *p* < 0.001) and 5xSTS (*β* = 0.38, *p* < 0.001) tests significantly increased with age. The prevalence of physical inactivity, low handgrip strength, poor 5xSTS performance, slow gait speed, TUG deficits, and possible sarcopenia significantly increased with age (*p* < 0.05, for all); however, the prevalence of comorbidities and falls did not significantly change with age.

### 3.2. Associated Factors of Possible Sarcopenia

Table 2 shows factors associated with possible sarcopenia using logistic regression, and indicated that age, physical inactivity, slow gait speed, and TUG deficits were significantly associated with possible sarcopenia. After adjusting for potential confounders, only age (adjusted OR 1.09, 95% CI 1.04–1.14), slow gait speed (adjusted OR 4.92, 95% CI 2.34–10.33), and TUG deficits (adjusted OR 6.65, 95% CI 3.20–13.84) were independently associated with possible sarcopenia.

### 3.3. Associated Factors of Physical Inactivity

Table 3 shows factors associated with physical inactivity using logistic regression, and indicated that age, low handgrip strength, poor 5xSTS performance, slow gait speed, and TUG deficits were significantly associated with physical inactivity. After adjusting for potential confounders, only low handgrip strength (adjusted OR 2.31, 95% CI 1.12–4.74), poor 5xSTS performance (adjusted OR 2.27, 95% CI 1.15–4.47), slow gait speed (adjusted OR 2.31, 95% CI 1.16–4.61), and TUG deficits (adjusted OR 2.87, 95% CI 1.44–5.73) were independently associated with physical inactivity.

## 4. Discussion

To our knowledge, this is the first study conducted at rural community daycare stations in Northern Hualien, Taiwan to examine the prevalence of and associated factors for physical inactivity and possible sarcopenia in adults aged ≥55 years, and to explore the relationship between physical inactivity and possible sarcopenia. Our findings do not support the hypothesis that physical inactivity may positively correlate with possible sarcopenia. Consistent with a previous cross-sectional study of Miura et al. (2021) using univariate regression analyses [17], our results showed that physical inactivity was positively correlated with possible sarcopenia (OR 3.49, 95% CI 1.77–6.89, *p* < 0.001). However, after adjusting for potential confounders, our results were incongruent with those of Miura et al. (2021), i.e., physical inactivity was not independently associated with possible sarcopenia. The disparity in correlation between possible sarcopenia and physical inactivity reflects differences in adjusting for covariates in multiple regression analyses. In the study of Miura et al. (2021) [17], hemoglobin A1c and oral function were used as covariates; however, in the present study, age, sex, and poor lower-limb muscle function (i.e., slow gait speed and TUG deficits) were used as covariates. Furthermore, evidence suggests that aerobic exercise had less impact on muscle mass or muscle strength than resistance exercise [31]. In the present study, only aerobic exercise was utilized to assess physical activity levels. According to AWGS 2019 criteria, possible sarcopenia is defined as low muscle strength and/or poor 5xSTS performance, both of which are of more relevance to resistance exercise than to aerobic exercise. Therefore, an aerobic-based physical activity level might not completely reveal the real association between physical inactivity and possible sarcopenia [9]. Another explanation is that a decline in muscle function during possible sarcopenia might not be enough to limit daily physical activity. Hence, the habits of engaging in daily physical activity were still retained, including walking and simple farming activities. In fact, our results show about two-thirds of adults with possible sarcopenia were still physically active.

It is worthy of note that, after adjusting for potential confounders, poor lower-limb muscle function, e.g., slow gait speed, was highly associated with both possible sarcopenia and physical inactivity. A population-based prospective cohort study has indicated that slower gait speed was associated with physical inactivity independently of osteoarthritis and handgrip strength [32]. Moreover, our results are congruent with a longitudinal study in older Chinese adults [16], indicating that slow gait speed was significantly associated with possible sarcopenia. The mechanisms by which slow gait speed is associated with physical inactivity and possible sarcopenia potentially involve complex relationships between motor and sensory processes in the brain [33]. Physical inactivity may increase muscle loss in the lower limb with aging [34], which could then affect gait mechanics [35] and decrease 5xSTS performance. Given that poor 5xSTS performance has been adopted to determine possible sarcopenia according to the AWGS 2019 criteria [4]. Therefore, our findings highlight that improving lower-limb muscle function might be a priority and an effective strategy for increasing physical inactivity and preventing or delaying possible sarcopenia in adults at rural community daycare stations.

Additionally, low BMI has been reported to be one of the risk factors for possible sarcopenia [17]. However, in the present study, linear regression analyses showed that BMI was not significantly associated with possible sarcopenia. One plausible explanation is that the mean BMI of 26.9 kg/m^2^ in the present study was greater than that of 19.4 kg/m^2^ in the study of Miura et al. (2021) for older adults with possible sarcopenia [17]. Previous studies indicated that higher BMI had a protective effect against possible sarcopenia [9,16,18]. Furthermore, our findings seem to echo a cross-sectional study of older adults in Switzerland by Bertschi et al. (2021), indicating that low BMI (with a mean of 25.6 kg/m^2^) was not associated with probable sarcopenia according to new criteria of the European Working Group on Sarcopenia in Older People 2 [36].

In the present study, we found that possible sarcopenia was highly prevalent in 68.7% of adults at rural community daycare stations. Our findings were inconsistent with two previous studies: one was conducted by Miura et al. (2021), which recruited older Japanese adults aged 60 years who underwent health and frailty checkups, with an estimated prevalence of 2.9% [17]; the other was conducted by Chang et al. (2021), which recruited older Taiwanese adults aged over 65 years (mean age 81.6 years) at daycare stations in Keelung City of Taiwan, with an estimated prevalence of 98.3% [18]. First, the difference was probably due to different operational definitions used to define possible sarcopenia. In the study of Miura et al. (2021), only muscle (handgrip) strength was applied as the criteria for diagnosis of possible sarcopenia [17]. Second, the differences may be ascribed to the age of the sample. In the present study, the mean age of the sample (71.9 years old) was older than that in the study of Miura et al. (2021) [17] and younger than that in the study of Chang et al. (2021) [18]. Previous studies have suggested that older age groups were more likely to develop possible sarcopenia [9,13,16]. Finally, the differences may be ascribed to the varied settings across studies. A previous study indicated that the prevalence in daycare stations was higher than that in the general community and close to that in clinical settings, and that the prevalence varied from 27.3% to 80.0% across different daycare stations [18].

Inconsistent with previous studies on the prevalence of possible sarcopenia in urban areas using the AWGS 2019 criteria [10,16,17,37,38], our results showed possible sarcopenia being highly prevalent in rural community daycare stations (68.7% vs. 2.9%–42%). One plausible explanation is an imbalanced healthcare resource between urban and rural areas. A nationally representative screening of the Chinese population aged ≥ 60 years indicated that older adults with possible sarcopenia in rural areas had a higher prevalence than those in urban areas [16]. Another explanation is the different representative samples studied. Compared to nationally representative screening [10,16] and large-scale health checkups [17], our study targeted a specific population of those receiving LTC services at rural community daycare stations. Individuals who were eligible to enter community daycare stations were more likely to have low muscle strength or poor physical performance because of frailty, disabilities, and other health conditions. Moreover, owing to the lack of appropriate diagnostic tools for measuring muscle mass in community settings to rule out confirmed sarcopenia and severe sarcopenia, our results may overestimate the prevalence of possible sarcopenia in rural community daycare stations. A recent cross-sectional study by Chang et al. (2021), conducted at eight community daycare stations in Keelung City, Taiwan [18], reported 98.3% of older adults with possible sarcopenia or confirmed sarcopenia. Compared with the results of Chang et al. (2021), the disparity in prevalence might reflect age (mean 71.9 vs. 81.6 years) and locality (rural vs. urban) differences.

In the present study, we found that 29.1% of adults aged ≥ 55 years in rural community daycare stations were physically inactive. Our results are incongruent with data from urban areas in Japan [17] and Thailand [37], which, respectively, reported 31.3% and 33.3% of older adults being physically inactive, both of which are higher than our findings from rural areas. One plausible explanation is occupational and lifestyle differences between rural and urban areas [39]. In Taiwan, most individuals in rural areas are engaged in heavy labor, while with advanced age, some older adults still remain in farming habits and continue to engage in simple farming activities. In fact, the rural–urban differences in physical inactivity were also observed in a nationally representative sample of the Chinese general population [39], revealing that physical inactivity was more common among populations in urban areas compared with those in rural areas. Furthermore, using univariate regression analyses, we found that physical inactivity rose with age in individuals at rural community daycare stations, which is consistent with a global report on physical activity levels [40]. However, after adjusting for potential confounders, only poor lower-limb muscle function, such as slow gait speed and TUG deficits, rather than age and upper-limb muscle function, was independently associated with physical inactivity. Our findings are consistent with the results of a previous study [41], reporting that poor lower-limb muscle function was associated with lower levels of physical activity in older adults. As mentioned above, some Taiwanese rural older adults still remain in farming habits, which could explain why the age was less likely to influence physical activity in our sample. Although our results show that poor lower-limb muscle function was associated with physical inactivity, poor lower-limb muscle function was highly prevalent compared to physical inactivity (41.5–50.2% vs. 29.1%). Hence, our result highlight that improving lower-limb muscle function should be a priority for adults in community daycare centers even though they reached the recommended physical activity levels.

Interestingly, according to the AWGS 2019 guidelines [4], low handgrip strength and poor 5XSTS performance have been proposed for identifying possible sarcopenia; however, both had different impacts on detecting the prevalence of probable sarcopenia. In the present study, we found that the prevalence of possible sarcopenia was 68.7%, of which 86.8% was due to poor 5xSTS performance and 57.7% to low handgrip strength, i.e., poor 5xSTS performance was dominant for detecting probable sarcopenia. Our findings are consistent with a nationwide cohort study in Korean community-dwelling older adults [10] and a cross-sectional study in Taiwanese older adults at urban community daycare stations [18], reporting that the prevalence of poor 5xSTS performance was higher than low handgrip strength. Because the 5xSTS test is a simple screening tool, it might deserve a first line screening tool for detecting most individuals with possible sarcopenia, especially in a limited-resource rural community setting. In addition, the 5xSTS performance relies more on lower extremity muscle strength [21], lower extremity muscle strengthening programs should be a priority for reducing the prevalence of possible sarcopenia. It is worth note that, compared to the old AWGS 2014 criteria, adding poor 5xSTS performance to the updated AWGS 2019 criteria may lead to more individuals being recognized as having possible sarcopenia; however, it is unclear yet whether this change is more likely to predict sarcopenia development [42], which requires more longitudinal studies to validate.

A previous cross-sectional survey showed that older adults were prone to disengage from physical activity after experiencing a fall [43]. Interestingly, in the present study, we found that a history of falls was not significantly associated with physical inactivity. Our results seem to echo a cross-sectional study in Malaysia reporting that physical activity level was not relevant to the risk of falls in community-dwelling older adults [44]. One plausible explanation is that physical inactivity may be related to fear of falling, but not to actual falls. In fact, a cross-sectional study has indicated that physical inactivity was not associated with actual falls, and those with a fear of falls were more likely to be physically inactive [43]. Furthermore, evidence suggests that older adults with sarcopenia have a high risk of falls [9]; however, few studies have explored the relationship between possible sarcopenia and falls. In the present study, we did not find a significant association between a history of falls and possible sarcopenia. One probable explanation is that possible sarcopenia and confirmed sarcopenia are at different stages of disease progression and might present different clinical outcomes. Another explanation is the benefits of widespread public health education on falls prevention [45]. In Taiwan, public health education is one of the most important services at community daycare stations. Considering that this is the first report to disclose the relationship between possible sarcopenia and falls at rural community daycare stations in Taiwan, and also falls are multifactorial and complex [46,47], future studies are required to confirm our findings.

This study has several strengths. The first is the novelty of examining the effects of an updated AWGS 2019 criteria, i.e., low handgrip strength, poor 5xSTS performance, or both, on the prevalence of and associated factors for possible sarcopenia in rural community daycare stations. Hence, our results can easily be compared with other Asian countries, and could also impact future LTC policy reforms and practices in Taiwan. Another strength is that all of the rural community daycare stations in Northern Hualien, Taiwan were sampled. Hence, our results represent the populations receiving LTC services at all rural community daycare stations across the region. However, some limitations are known. First, adults aged ≥ 55 years and receiving LTC services at rural community daycare stations in Northern Hualien, Taiwan were recruited. Thus, we caution against generalizing our findings to adults aged < 55 years or those dwelling in other areas of Taiwan, in institutional locales, or in general communities that are not receiving community daycare services. Second, there was an uneven ratio of eligible men and women, with most participants being female in community daycare stations, preventing exclusion of a potential confounding sex effect. Some studies indicated that age-related hormonal changes had a positive impact on muscle mass and strength in women, but not in men [48,49]. However, Taiwanese older women have larger populations, longer life expectancy, and higher rates of disability [1], and thus more of them are eligible for availability of LTC services in their communities than older men [18,50]. In fact, a similar phenomenon can also be observed in a previous study in Keelung City, Taiwan, showing a high ratio of eligible women (75.3%) in community daycare stations [18]. Third, some case-finding tools for sarcopenia, such as calf circumference and questionnaires, were not used in the present study. Although these case-finding tools were recommended by AWGS 2019, these have been reported to be of low sensitivity for screening sarcopenia [51,52,53]. Finally, this was a cross-sectional study, and causality is not well established. However, since possible sarcopenia has been considered an initial manifestation of sarcopenia [4,21], identifying the associated factors for possible sarcopenia can further help prevent sarcopenia development in rural community daycare stations.

## 5. Conclusions

Our results demonstrate a novel result, showing that physical activity was not independently associated with possible sarcopenia in rural community daycare stations. By contrast, poor lower-limb muscle function and strength, e.g., slow gait speed and poor 5xSTS performance, were independently associated with both possible sarcopenia and physical inactivity. Given that about 86.8% of possible sarcopenia can be identified by poor 5xSTS performance, more attention should be paid to the assessment of 5XSTS performance and to the intervention of lower-limb muscle function for individuals in community daycare stations. It required future studies to confirm our findings using a larger, more balanced district sample, such as those in urban and suburban areas.

## Figures and Tables

**Table 1:** Participant characteristics and their relationship with advanced age.

Characteristics	All (*n* = 275)	Men (*n* = 69, 25%)	Women (*n* = 206, 75%)	Regression Analysis with Advanced Age ^a^		
				B	*β* or EXP (B)	*p* Value
Age (years)	71.9 (70.8–73.0)	73.2 (70.8–75.5)	71.5 (90.2–72.8)			
BMI (kg/m^2^)	26.9 (26.3–27.6)	25.9 (24.7–27.1)	27.3 (26.6–28.0)	−0.10	−0.19	**0.003**
Comorbidities, *n* (%)						
Hypertension	153 (55.4%)	32 (46.4%)	121 (58.7%)	−0.00	1.00	0.893
Heart disease	46 (16.7%)	10 (14.5%)	36 (17.5%)	0.01	1.01	0.675
Diabetes mellitus	82 (29.7%)	19 (27.5%)	63 (30.6%)	−0.00	1.00	0.923
Knee Osteoarthritis	48 (17.5%)	3 (4.3%)	45 (21.8%)	0.00	1.00	0.938
Stroke	20 (7.3%)	6 (8.7%)	14 (6.8%)	0.00	1.00	0.946
History of falls, *n* (%)	86 (31.3%)	22 (31.9%)	64 (31.1%)	0.01	1.01	0.655
Physical inactivity, *n* (%)	80 (29.1%)	15 (21.7%)	65 (31.6%)	0.05	1.05	**0.001**
Handgrip strength (kg)	21.5 (20.7–22.4)	27.2 (25.1–29.3)	19.6 (18.9–20.3) *	−0.27	−0.35	**<0.001**
5xSTS (s)	15.0 (13.9–16.0)	16.5 (14.4–18.7)	14.5 (13.3–15.7)	0.36	0.38	**<0.001**
Gait speed (m/s)	1.00 (0.96–1.04)	0.92 (0.84–0.99)	1.04 (0.99–1.08) *	−0.02	−0.39	**<0.001**
TUG (s)	13.3 (12.2–14.5)	14.6 (12.7–16.4)	12.9 (11.5–14.4)	0.28	0.26	**<0.001**
Low handgrip strength ^b^, *n* (%)	109 (39.6%)	38 (55.1%)	71 (34.5%) *	0.11	1.11	**<0.001**
Poor 5xSTS performance ^c^, *n* (%)	164 (59.6%)	46 (66.7%)	118 (57.3%)	0.08	1.09	**<0.001**
Slow gait speed ^d^, *n* (%)	114 (41.5%)	35 (50.7%)	79 (38.3%)	0.09	1.09	**<0.001**
TUG deficits ^e^, *n* (%)	133 (48.4%)	45 (65.2%)	88 (42.7%) *	0.10	1.11	**<0.001**
Possible sarcopenia, *n* (%)	189 (68.7%)	54 (78.3%)	135 (65.5%) *	0.11	1.12	**<0.001**

Values are presented as mean (95% CI), or frequencies (percentages). BMI, body mass index; 5xSTS, five-times-sit-to-stand test; TUG, timed up-and-go test. ^a^ Estimated coefficient for linear regression (continuous variables) or logistic regression (categorical variables). ^b^ Defined as low handgrip strength < 28 kg and <18 kg for men and women, respectively. ^c^ Defined as poor 5xSTS performance > 12 s. ^d^ Defined as slow gait speed < 1.0 m/s. ^e^ Defined as TUG deficits > 11 s. Bold indicates significance with *p* < 0.05. * indicates significant gender difference (at *p* < 0.05).

**Table 2:** Factors associated with possible sarcopenia using multiple logistic regression.

Variables	Model 1			Model 2		
	Crude OR	95% CI	*p*-Value	Adjusted OR	95% CI	*p*-Value
Age (years)	1.12	1.08–1.16	<0.001	1.09	1.04–1.14	**<0.001**
Sex (with women as ref.)	1.89	1.00–3.59	0.051			
BMI (kg/m^2^)	0.98	0.93–1.03	0.392			
Number of comorbidities	1.03	0.80–1.33	0.825			
Medical conditions						
Hypertension	0.85	0.50–1.43	0.846			
Heart disease	1.23	0.61–2.47	0.569			
Diabetes mellitus	1.41	0.79–2.51	0.244			
Osteoarthritis	0.92	0.47–1.79	0.803			
Stroke	0.86	0.33–2.23	0.751			
History of falls	1.38	0.78–2.43	0.264			
Physical inactivity	3.49	1.77–6.89	**<0.001**	1.95	0.88–4.30	0.100
Slow gait speed (<1.0 m/s)	8.17	4.08–16.36	**<0.001**	4.92	2.34–10.33	**<0.001**
TUG deficits (>11 s)	11.49	5.83–22.67	**<0.001**	6.65	3.20–13.84	**<0.001**

BMI, body mass index; TUG, timed up-and-go test; OR, odds ratio; CI, confidence interval. Model 1, no adjustment; Model 2, adjusted by sex, physical inactivity, slow gait speed, and TUG deficits for age; adjusted by age, sex, slow gait speed, and TUG deficits for physical inactivity; adjusted by age, sex, physical inactivity for slow gait speed and TUG deficits. Bold indicates significance with *p* < 0.05.

**Table 3:** Factors associated with physical inactivity using multiple logistic regression.

Variables	Model 1			Model 2		
	Crude OR	95% CI	*p*-Value	Adjusted OR	95% CI	*p*-Value
Age (years)	1.05	1.02–1.08	**0.001**	1.00	0.96–1.04	0.875
Sex (with women as ref.)	0.61	0.32–1.17	0.138			
BMI (kg/m^2^)	1.04	0.99–1.10	0.147			
Number of comorbidities	1.29	1.00–1.67	0.051			
Medical conditions						
Hypertension	1.14	0.67–1.96	0.624			
Heart disease	1.51	0.77–2.94	0.277			
Diabetes mellitus	1.16	0.66–2.04	0.605			
Osteoarthritis	1.56	0.81–3.01	0.184			
Stroke	1.02	0.38–2.77	0.963			
History of falls	1.26	0.73–2.19	0.408			
Low handgrip strength	3.27	1.91–5.61	**<0.001**	2.31	1.12–4.74	**0.023**
Poor 5xSTS performance (≥12 s)	2.90	1.61–5.21	**<0.001**	2.27	1.15–4.47	**0.018**
Slow gait speed (<1.0 m/s)	3.95	2.28–6.85	**<0.001**	2.31	1.16–4.61	**0.018**
TUG deficits (>11 s)	4.09	2.32–7.23	**<0.001**	2.87	1.44–5.73	**0.003**

BMI, body mass index; 5xSTS, five-times-sit-to-stand test; TUG, timed up-and-go test; OR, odds ratio; CI, confidence interval. Model 1, no adjustment; Model 2, adjusted by the factors that showed *p*-values ≤ 0.2 in the univariate regression analyses: age, sex, BMI, number of comorbidities, osteoarthritis, low handgrip strength, and poor lower-limb muscle function (i.e., poor 5xSTS, slow gait speed, and TUG deficits). Bold indicates significance with *p* < 0.05.

## Data Availability

The datasets generated during and analyzed during this study are available from the corresponding author on reasonable request.
